# Supplementary material for: Evaluation of Novel B1R/B2R Agonists Containing TRIOZAN™ Nanoparticles for Targeted Brain Delivery of Antibodies in a Mouse Model of Alzheimer Disease
Source: Molecules. 2023 Jul 4;28(13):5206. doi: 10.3390/molecules28135206 (PMC10343654; doi:10.3390/molecules28135206)
Supplement: Supplementary file 1 [file molecules-28-05206-s001.zip › molecules-2474905-supplementary.pdf]

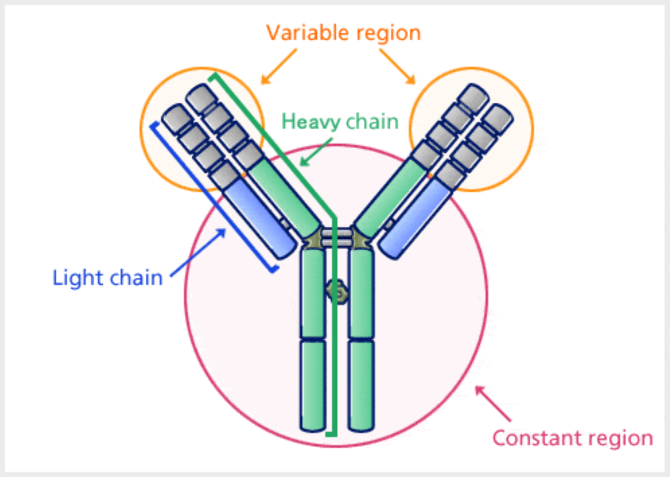

Heavy chain (51.15 kDa)

MDRLTSSFLLLIVPAYVLSQVQLQQPGAELVKPGASVKLSCKSSGYHFTSYWMHWVKQRPGQGLEWIGVIHPNSGSIN
YNEKFESKATLTVDKSSSTAYMQLSSLTSEDSAVYYCAGERDSTEVLPMDYWGQGTSVTVSSAKTTPPSVYPLAPGSAA
QTNSMVTLGCLVKGYFPEPVTVTWNSGSLSSGVHTFPAVLESDLYTLSSSVTVPSSPRPSETVTCNVAHPASSTKVDKKI
VPRDCGCKPCICTVPEVSSVFIFPPKPKDVLTITLTPKVTCVVVDISKDDPEVQFSWFVDDVEVHTAQTQPREEQFNSTF
RSVSELPIMHQDWLNGKEFKCRVNSAAFPAPIEKTISKTKGRPKAPQVYTIPPPKEQMAKDKVSLTCMITDFFPEDITVE
WQWNGQPAENYKNTQPIMNTNGSYFVYSKLNVQKSNWEAGNTFTCSVLHEGLHNHHTEKSLSHSPGK

Light chain (25.92 kDa)

MRFQVQVLGLLLLWISGAQCDVQITQSPSYLAASPGETITINCRASKSINKYLAWYQEKPGKTNKLLIYSGSTLQSGIPSR
FSGSGSGTDFTLTISSLEPEDFAMYYCQQHNEYPLTFGAGTKLELKRADAAPTVSIFPPSSEQLTSGGASVVCFLNNFYPK
DINVKWKIDGSERQNGVLNSWTDQDSKDSTYSMSSTLTLTKDEYERHNSYTCEATHKTSTSPIVKSFNRNEC

Supplementary Figure S1. Schematic representation and amino acid sequence of the mAbs ANX-M1. The selected peptide sequence for the quantitative LC-MS/MS analysis of ANX-M1 is indicated in green.

F4. TMC-B1R-DS-TPP (mAb + B2R agonist)

Z-ave = 165.6 ± 5.5 nm

PdI = 0.213 ± 0.004

Dh (number mean) = 69.4 ± 15.3 nm

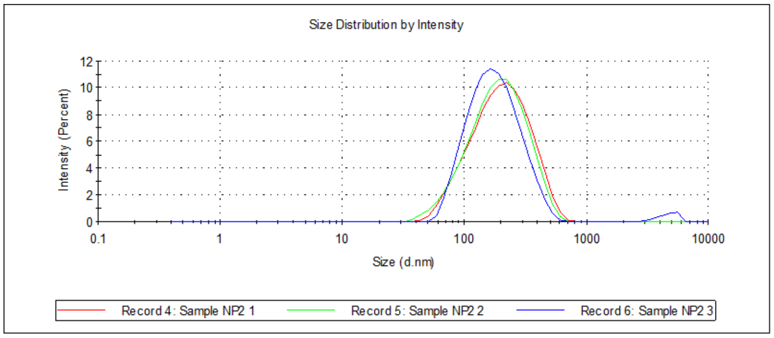

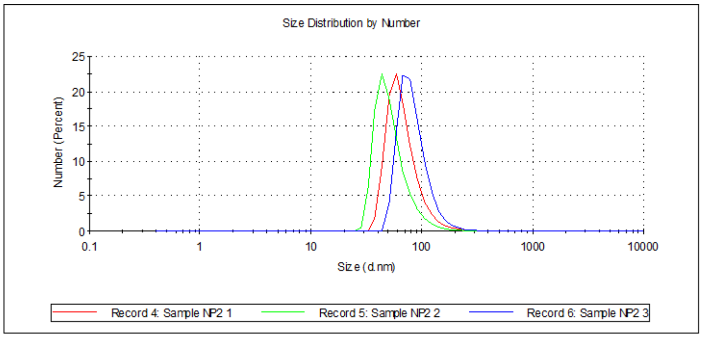

F6. TMC-DS-TPP (mAb + B1R + B2R)

Z-ave = 132.4 ± 2.3 nm

PdI = 0.144 ± 0.006

Dh (number mean) = 79.2 ± 7.3 nm

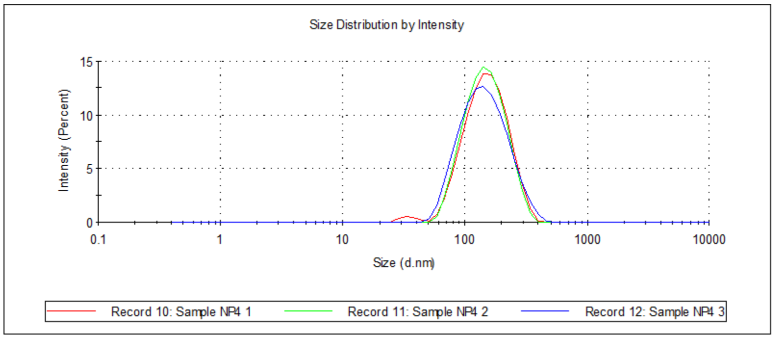

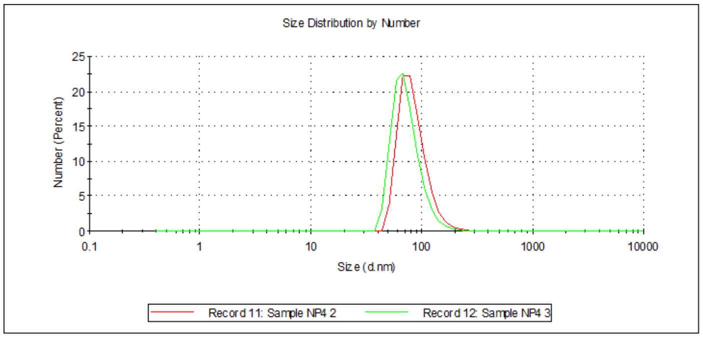

Supplementary Figure S2. Representative DLS graphs for the nanoformulations F4 and F6.
